# Reply letter to Dr. Wojtasek regarding: Oxidation of flavonoids by tyrosinase and by o-quinones–comment on "Flavonoids as tyrosinase inhibitors in *in silico* and *in vitro* models: basic framework of SAR using a statistical modelling approach” published by K. Jakimiuk, S. Sari, R. Milewski, C.T. Supuran, D. Söhretoglu, and M. Tomczyk (J Enzyme Inhib Med Chem 2022;37:427-436)

**DOI:** 10.1080/14756366.2023.2269613

**Published:** 2023-10-18

**Authors:** Michał Tomczyk, on behalf of all co-authors

**Affiliations:** Department of Pharmacognosy, Faculty of Pharmacy with the Division of Laboratory Medicine, Medical University of Bialystok, Białystok, Poland

Responding to the observations made by Dr. Wojtasek, we would like to evidence the relevant differences between out experimental conditions^1^ and those described in the papers by Dr. Wojtasek’s group^2^^,^^3^. In their experiments, Dr. Wojtasek and co-authors worked with the same substrate (DOPA) and inhibitor concentration, of 50 µM^2^^,^^3^. In our study^1^, similar to many other publications in the field^4^^,^^5^, the concentration of DOPA (the enzyme substrate) was of 0.25 − 2 mM, which is 5–40 times higher than the one of the works by Gąsowska-Bajger and Wojtasek^2^^,^^3^, leading thus to a saturation of the tyrosinase active site with the substrate and not allowing a competition between the enzyme and the inhibitor (present at equimolar concentrations, as in the situation described in Dr. Wojtasek’s work^2^^,^^3^. Furthermore, these authors worked with an enzyme concentration of 55 EU/mL, whereas in our study^1^ the tyrosinase concentration was of 250 EU/mL. These differences in enzyme and substrate concentrations between the two studies make the experimental conditions considerably different, also considering the fact that we tested the inhibitors from nanomolar to millimolar concentrations^1^.

It may be possible that under the experimental circumstances used by Gąsowska-Bajger and Wojtasek^2^^,^^3^ where the substrate and inhibitor concentrations were equivalent, a competition between the inhibitor and the substrate for binding within the enzyme active site may occur, which might induce inhibitor oxidation, as described by Gąsowska-Bajger and Wojtasek^2^^,^^3^. However, under conditions used in our experiments^1^, the substrate concentration was orders of magnitude higher than that of the inhibitor, making the oxidation unlikely, also because the enzyme active site was saturated with substrate (L-DOPA)^1^.

We found the comment regarding the use of thematic database confusing. There are many tyrosinase studies available in the literature using the same enzyme inhibition assay – considered to be the gold standard – as we did in our study^1^. It should be also mentioned that our study^1^, apart from measuring inhibition with flavonoids (which in fact were reported earlier as tyrosinase inhibitors [4]) was mainly a computational one which aimed to explain the binding mode of such compounds within the enzyme active site, considering the fact that few (and just recent) crystallographic studies of this enzyme are available^5^.
